# Attention in laboratory tasks versus naturalistic learning contexts: Heterogeneity in measurement and mechanisms

**DOI:** 10.21203/rs.3.rs-9511914/v1

**Published:** 2026-05-21

**Authors:** Fang Yu Chang, Xinyi Zoe Mao, Maya Khalil, Mark D. Rapport, Andrea Dillon, Sandra K. Loo, Jennie K. Grammer, Agatha Lenartowicz

**Affiliations:** University of California, Los Angeles; University of California, Los Angeles; University of California, Los Angeles; University of Central Florida; University of California, Los Angeles; University of California, Los Angeles; University of California, Los Angeles; University of California, Los Angeles

**Keywords:** EEG, Neural Oscillations, Alpha, Attention, Classroom, Learning

## Abstract

An important though less studied element in neuroscience of classroom learning, is translation of laboratory-derived metrics to quantify learning in naturalistic settings. In the current study we sought to test if a common neural metric of visual attention, alpha-range (8–12 Hz) oscillations, correlates across laboratory versus naturalistic learning activities. Thus, we test if laboratory-derived measures capture the same mechanistic sources when deployed in more naturalistic environments. We acquired electroencephalography signals in eighty 6–10-year-old children while they participated in naturalistic learning activities and during a laboratory spatial working memory task. We found that alpha power modulations during the activities did not correlate with modulations during stimulus processing in the laboratory task, despite (i) significant correlations across baseline periods of each context, (ii) internal consistency across contexts in the relationship of baseline and modulation effects, and (iii) significant association with behavioral indicators of attention. The results point to measurement heterogeneity across contexts, consistent with different mechanisms contributing to these metrics during learning activities versus laboratory task. The results have implications for experimental design in the domain of education neuroscience and argue for consideration of multiple processes – such as multisensory processing, arousal, motivation, and motor activity – when interpreting students’ observable behavior.

## Introduction

Translation from laboratory to real-world environments is a prime objective in neuroscience but is not always achieved. This is especially notable in neurodevelopmental research. For instance, in the case of attention deficit hyperactivity disorder (ADHD), characterized by symptoms of inattention and hyperactivity^1^, impacted children are more likely to be placed in special classes (10–30% vs <5%), repeat grades (20–40% vs <10%), drop out of high-school/college (10–50% vs <10% nationally^2^) and less likely to pursue post-high-school education (73.1% vs 95.1%)^3–5^{Barkley, 1990 #9158;Fischer, 1990 #9157;Barkley, 2006 #8538}. Notably, weaker academic attainment predicts job loss in this population^6^{Kuriyan, 2013 #9167}. Yet laboratory assessments of inattention used in studies of ADHD have weak translational impact^7,8^. For instance, performance on the continuous performance task, commonly used to assess attention deficits, fails to predict in-classroom observations^9^ and/or shows weak correlations (.25–.35)^7^, highlighting the need for translation-focused paradigms.

This gap in translation has motivated efforts to measure attention in more naturalistic settings, enabling researchers to examine how environmental context, interactions between students and educators, and student-level individual differences in classroom shape attention during learning *in situ*. Mobile electroencephalography (EEG) has emerged in the past decade as a promising tool for objective quantification of attention^10^, with increasing application in the educational domain in studies of attention, engagement, cognitive load, and related factors, and across the developmental trajectory from kindergarten to university^11,12^. Objective indicators of attention processes are most commonly derived from the spectral content of the continuous EEG signals recorded during learning activities, such as power of theta (4–7 Hz), alpha (8–12 Hz), and beta (13–30Hz) band oscillations, as well as derived measures such as ratios^11^. For instance, changes in power of occipital alpha oscillations, during naturalistic learning have shown that interactive activities, such as group and independent work in college students^13^, or hands-on activity in elementary school students^14^, are more attentionally engaging than asynchronous learning activities such as watching an educational video.

However, the use of such neural measures in natural contexts has raised a new question: whether the mechanistic inferences associated with a given measure are the same in the naturalistic environment as in the laboratory context in which those measures were originally developed. For instance, power decreases in the above-noted alpha oscillations have been associated with greater visuo-spatial attention processing and selective attention^15–17^, leading to the inference that modulations of alpha power reflect modulations in visual attention. Most of this evidence, however, comes from event-related experimental designs, which effectively isolate a connection between the decrease in alpha power with, for instance, enhanced visual processing relative to a pre-stimulus baseline. This is called an event-related modulation (ERM). However, in naturalistic environments, such event-related isolation may not be possible, and alpha power is calculated across a window that encompasses many events (i.e., often called absolute alpha power). This is potentially problematic because alpha oscillations can also respond to processes such as memory retrieval, response generation and changes in arousal^18^, implying that, in naturalistic environments the feature is potentially subject to heterogeneity in sources that drive variability in observed signal.

In the current study we thus sought to test if alpha oscillations collected during lab tasks, a robust indicator of visuospatial system engagement, predict individual differences in alpha oscillation measures collected in the same children during naturalistic learning activities designed to approximate classroom contexts. To examine this, we calculated both event-related modulations and absolute power derivatives of the neural measures across testing contexts to establish if these measures correlate across individuals, thus testing if they capture the same mechanistic sources or if they are subject to measure heterogeneity with change in context. The study sought to inform if and how neural features should be deployed in studies of neural processes during naturalistic earning to optimize mechanistic interpretability.

## RESULTS

**Figure 1:**
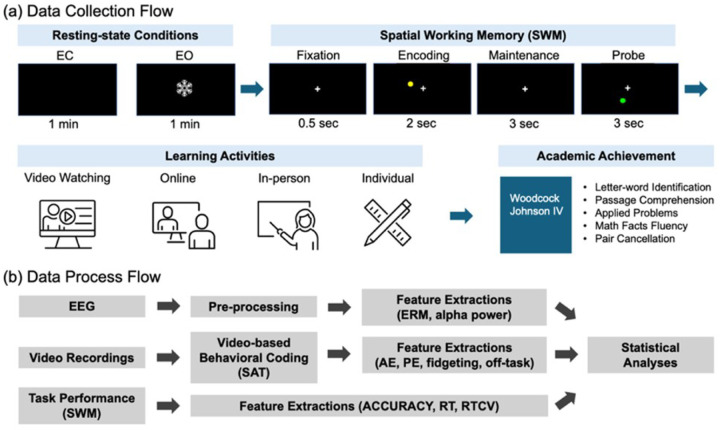
Session scheme and analysis pipeline. (a) Each session began with resting-state recordings (eyes closed “EC”, eyes open “EO”; 1 min each), followed by a spatial working memory (SWM) task including fixation (0.5 s), encoding (2 s), maintenance (3 s), and probe (3 s). Memory load was varied by the number of dots during encoding (1, 3). Participants then completed classroom learning activities (video watching, online teacher instruction, in-person teacher instruction, individual learning; about 10 min each) and academic assessments from the Woodcock-Johnson (IV) academic achievement test. (b) Event-related modulation (ERM) and absolute alpha power was extracted from EEG recordings. Video recordings were coded using the Student Attention Tracking (SAT) protocol to derive attention engagement and fidgeting measures. SWM performance metrics included accuracy, reaction time (RT) and its variability (RTCV). Icons in the Learning Activities panel were adapted from Adobe Stock.

Eighty elementary school children (6 to 10 years old) were enrolled to participate in the study. During the testing session (Fig. 1) they completed a laboratory task followed by naturalistic learning activities. The laboratory task was a spatial working memory (SWM) task^19^, shown to reliably modulate alpha power during stimulus processing, whereby decreases in alpha power reflect increases in visual engagement, and sensitivity to individual differences therein^20^. The naturalistic learning activities included four, ten-minute segments during which children were engaged in neuroscience-themed lessons in four activities (video watching, online teacher interaction, in-person teacher interaction, and hands-on individual learning) designed to test the effects of instructional context on engagement during learning^14^. We also obtained two baseline recordings with eyes open and eyes closed, to provide a reference for the magnitude of alpha oscillation effects. During all lab tasks and activities, EEG signals were continuously recorded using mobile EEG, and alpha-band (8–12Hz) oscillation power (reflecting the square of the magnitude) and its modulations were extracted. The core question of the study was whether individual differences in alpha power observed during the laboratory SWM task correlate with modulations observed during naturalistic learning activities.

### Alpha power decreases with visual engagement

**Figure 2:**
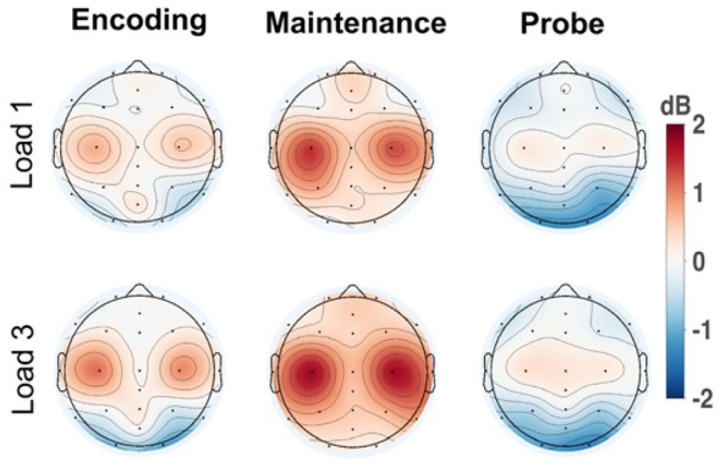
Topography of alpha event-related modulation (ERM) during encoding, maintenance, and probe phases. Alpha power decreased across posterior electrodes relative to pre-trial baseline during stimulus processing (encoding, probe) and increased during maintenance.

We first sought to validate that alpha power was reliably modulated within each recording context, given its proposed role in visuospatial system engagement^15,18^. During the lab SWM task, as shown in Figure 2, alpha power decreased across posterior electrodes during stimulus processing (mean_encoding_ = −0.257 dB, *p*_encoding_ = 0.072; mean_probe_ = −1.073 dB, *p*_probe_ < 0.001) relative to pre-trial baseline, consistent with visuospatial engagement. Also consistent with prior results, these stimulus-processing event-related modulations (ERMs) during encoding and probe were significantly correlated (*ρ*_encoding–probe_ = 0.769, *p* < 0.001). During maintenance, alpha power increased numerically relative to baseline, and this was significantly different than baseline (mean_maintenance_ = −0.309, *p*_maintenance_ = 0.045). Neither the main effect of load (i.e., number of dots in stimulus, c.f., Fig. 1, *F(1,48)* = 0.481, *p*= 0.491), nor trial phase (*F(2, 96)* = 1.071, p = 0.347), nor their interaction (*F(2, 96)* = 0.101, *p* = 0.904) was significant. Thus, relative to pre-stimulus baseline, alpha power showed only the expected ERMs relative to baseline, decreases in power associated with stimulus processing during encoding and probe, and increase during maintenance.

**Figure 3:**
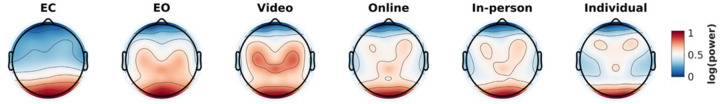
Topographical distribution of alpha power across resting-state conditions and learning activities. Alpha power was elevated during the resting state (EC, EO) relative to the learning activities, with a posterior scalp distribution. During the learning activities, alpha power was observed across both central and posterior electrodes, decreasing progressively from asynchronous (video watching) to teacher-led synchronous (online, in-person) to student-led (individual) activities.

Next, we tested whether absolute alpha power was also modulated during the learning activities. Across posterior electrodes, there was a significant main effect of activity (*F*(5, 240) = 4.758, *p* < 0.001), with alpha power decreasing from EC to EO resting-state conditions and furthermore across learning activities (from left to right in Figure 3). Pairwise comparisons indicated that alpha power was highest when the eyes were closed (EC) relative to eyes-open (EO) and all learning activities (*p* < 0.001), as expected with the elimination of visual input to the visual cortex. Alpha power was also higher in EO relative to all learning activities (*p* < 0.001), suggesting greater visual engagement during the activities. Among instructional contexts, alpha power was highest during video watching relative to the other activities (*p* < 0.001) and lowest during the individual activity relative to the other activities (*p* < 0.001). Alpha power did not significantly differ between the online and in-person conditions (*p* = 0.474). The stepwise decrease in alpha power across learning activities suggests a progressive increase in visual engagement from asynchronous (video watching) to teacher-led (online and in-person) to student-led (individual) contexts. In sum, in both lab task and learning activities alpha power decreases co-occurred with inferred increases in visual processing.

### Alpha power correlations between lab task and learning activities

**Figure 4:**
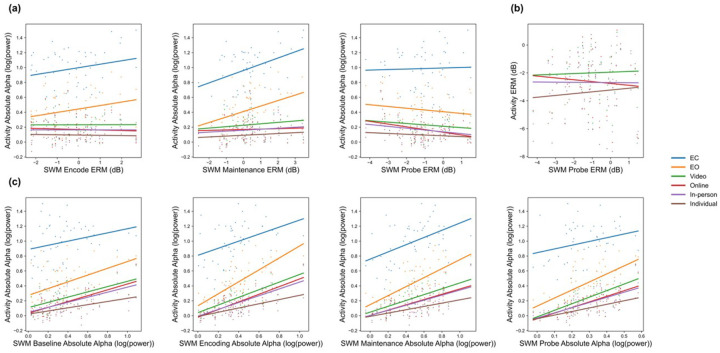
Associations between alpha power during SWM task measures and learning activities. Top Row: (a) No correlations were observed between alpha ERM in stimulus processing phases of the SWM task (encoding, probe) and absolute alpha power during learning activities (video, online, in-person, individual). Similarly, (b) correlations were not significant for EO-normalized ERM during activities relative to the SWM ERMs (probe shown, c.f., supplemental materials for full results), suggesting that the absence of correlations in (a) is not due to differences in measure derivation. Bottom Row: In contrast, (c) positive correlations were observed between absolute (non-ERM) SWM alpha power during each trial phase (baseline, encoding, maintenance, probe) and absolute alpha power during each learning activity. The results suggest that ERMs of alpha power during task capture different processes than modulations during the activities, unlike absolute SWM alpha power. Lines represent ordinary least squares linear fits.

Next, we sought to quantify associations between laboratory-derived and activity-based EEG measures. As shown in Figure 4a, event-related alpha modulations during lab task were overall not significantly correlated with absolute alpha power during learning activities across posterior electrodes. ERM during stimulus processing (encoding, maintenance or probe) was not significantly correlated with alpha power in EC, EO, video, online, in-person, or individual activities (*p*s > 0.05). These data indicate limited correspondence between ERM of alpha power during stimulus processing and absolute alpha power observed during learning activities. During the maintenance period, modest positive associations emerged. Specifically, ERM during maintenance was significantly correlated with alpha power during EC and EO (*ρ*_EC_ = 0.31, *p*_EC_ = 0.041; *ρ*_EO_ = 0.38, *p*_EO_ = 0.011). These findings suggest a selective correspondence between maintenance-related ERM and EC and EO alpha power, but not with activity alpha during learning activities.

The lack of correspondence between ERM in alpha power manipulated through a laboratory task and absolute alpha power observed during naturalistic learning activities may suggest that visual encoding processes captured by task ERMs are not the driving source behind individual differences in visual engagement during the activities. Given the alpha measure derivations differed between lab (ERM) versus activity (absolute alpha power), however, the lack of correlations could also be attributed to change in the derivation of the measure. To test this hypothesis, we normalized the absolute alpha power during activities relative to that in the EO condition (i.e., dividing activity power by EO power), creating an analogous ERM metric to that during visual processing in the lab task. However, this \ERM metric during activities remained statistically uncorrelated to encoding and probe ERMs (Fig. 4b, *p* > 0.05, c.f., supplemental materials for additional results). As such, the lack of correspondence between ERMs in alpha power during stimulus processing (SWM) versus during learning activities is unlikely to be due to differences in measure derivation.

Finally, we sought to confirm that individual differences in absolute alpha power, outside of ERMs, are preserved across laboratory and learning activity contexts. This would speak to the internal consistency of non-ERM alpha power indices within the experiment. Indeed, correlations in absolute, non-event related alpha power were significantly correlated between SWM trials and learning activities (Figure 4(c); ρ_baseline_ = 0.43–0.53, ρ_encoding_ = 0.58–0.72, ρ_maintenance_ = 0.54–0.63, ρ_probe_ = 0.65–0.78, *p*s < 0.001), suggesting that these non-ERM metrics reliably capture individual differences between lab and activity conditions. Interestingly, the only non-significant relationships were observed for alpha power during the EC condition in two of the four trial windows (ρ_EC-baseline_ = 0.27, *p*_EC-encoding_ = 0.063; ρ_EC-probe_ = 0.23, *p*_EC-probe_ = 0.109), which is consistent with a more significant change in visual input to the cortex, and thus potentially oscillatory state, when the eyes are closed.

### Correlation between baseline alpha power and event-related modulation.

**Figure 5:**
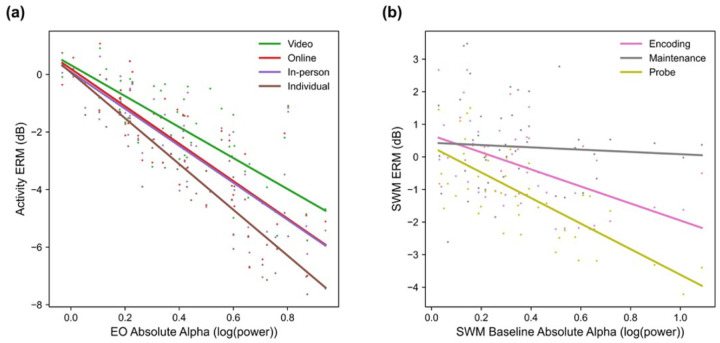
Alpha ERMs versus baseline power. (a) Associations between alpha power modulation (normalized to eyes-open, EO) during learning activities (video, online, in-person, individual) and reference (i.e., EO) alpha power. (b) Associations between SWM event-related modulation (ERM) and SWM baseline alpha power across task phases (encoding, maintenance, probe). Across lab and activities, greater baseline alpha power is associated with a greater decrease during ERM. Lines represent ordinary least squares linear fits.

**Figure 6:**
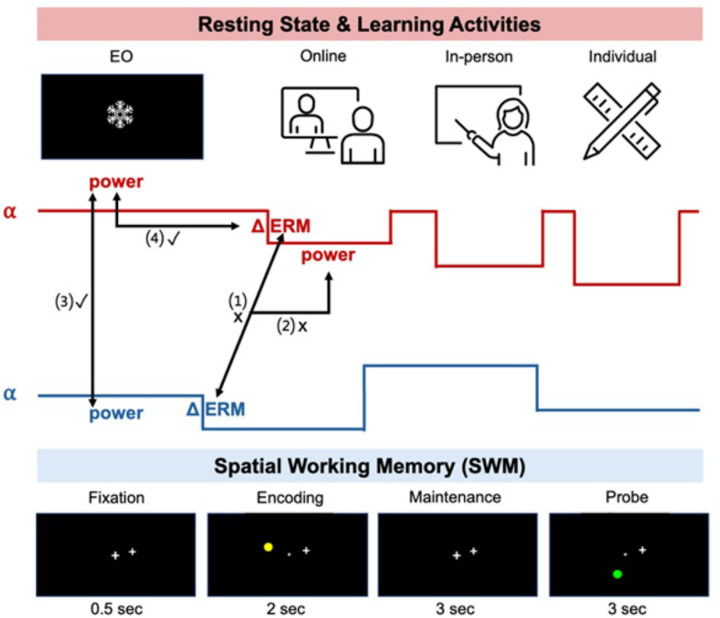
Summary of associations between alpha power and event-related modulation (ERM). Red and blue traces illustrate alpha power and task-related alpha modulation (ΔERM) measured during learning activities and the spatial working memory (SWM) task. Arrows indicate tested relationships between measures. Lab ERMs during stimulus processing were not correlated with (1) activity ERM, nor with (2) activity absolute alpha power. In contrast, (3) absolute alpha power during rest and baseline conditions was correlated across contexts, and (4) baseline absolute alpha power was correlated with alpha ERM within each context. The results indicate that while resting state alpha power captures individual differences reliably, ERMs and thus their underlying sources differ between task and activity modulations. Check marks indicate significant associations and crosses indicate non-significant associations.

The results thus far indicate that activity-related and lab task ERM of alpha power capture different sources of variance, presumably related to transient cognitive processing during the lab stimuli versus additional processes during activity periods (c.f., Fig. 6 for a summary). However, we were interested in also testing if absolute alpha power during a designated baseline period, during which cognitive processing is assumed to be at a rest state, is indicative of the amount of modulation observed during an active task state. This is important for establishing internal consistency between absolute and event-related measures within each recording context. As such, we correlated alpha power independently in the activity and lab task data during baseline with the ERMs. The baseline was designated as pre-trial in the SWM task, and as the EO condition in the activity context. As is shown in Figure 5, the relationship is consistent, with higher baseline alpha power associating with stronger decreases during active task periods (ρ_EO-Activity_ = −0.76–0.91, all *p*_EO-Activity_ < 0.001; ρ_baseline-encode_ = −0.53, *p*_baseline-encode_ < 0.001; ρ_baseline- maintenance_ = −0.04, *p*_baseline- maintenance_ = 0.760; ρ_baseline-probe_ = −0.67, *p*_baseline-probe_ < 0.001).

This finding is important for two reasons. It confirms that the dissociation between activity versus lab task ERM alpha power is limited to the “active” periods, and unlikely to reflect a difference in how the measure is calculated (absolute versus event-related). The finding also suggests that different sources contribute to the alpha signal during the cognitively active periods of the SWM task versus active learning. Additionally, the absence of a correlation only during the maintenance period of the SWM task (Fig. 5b, grey line) indicates that alpha power returns to baseline during maintenance period of the SWM task, consistent with its significant correlations with resting state (EC, EO) power (c.f., Fig. 4a).

### Alpha power prediction of outcomes: Lab task versus learning activity measures

Given the weak cross-measure correlations between task ERMs and activity alpha power, we hypothesized that the latter may capture different modulatory influences on visual attention (and therefore alpha power) than task stimulus-related ERMs. To explore this possibility, we tested whether these EEG features differ in relations to outcome variables. Separate multiple regression models were conducted for each behavioral dependent variable (for activity, video-coded behavior; for SWM task, performance), with ERM and absolute alpha power entered simultaneously, along with covariates of age, sex, and caregiver-reported inattention symptoms.

#### Laboratory Task Measures.

We first evaluated if EEG features (absolute alpha power and ERM during encoding, maintenance, and probe) predict either task performance or video-coded behavior in the laboratory SWM task (Table 1). As shown in Table 1, significant relationships to performance and behavior during task were primarily observed for ERM predictors. Specifically, more positive alpha ERM during probe predicted higher attention (*b*_attention_ = 0.504, *t*(40) = 2.719, *p* = 0.010) and lower fidgeting (*b*_fidgeting_ = −0.470, *t*(40) = −2.513, *p* = 0.016). Additionally, more negative alpha ERM during maintenance predicted higher accuracy (*b*_accuracy_ = −0.492, *t*(40) = −2.100, *p* = 0.042, and lower off-task behavior (*b*_off-task_ = 0.601, *t*(40) = 2.277, *p* = 0.028). The latter result is difficult to interpret, however, as the overall model fit was not significant when predicting off-task behavior. Unlike ERM predictors, absolute alpha power predictors were not related to behavioral outcomes. Taken together, it appears that absolute alpha power contributed less consistently to task performance and behavior compared to ERMs. ERMs showed significant associations with task accuracy and video-coded behavior (attention, fidgeting, off-task gaze/verbalizations), which is consistent with ERMs capturing transient cognitive processes related directly to task demands.

Finally, consistent with expectations, older age predicted higher accuracy (*b*_accuracy_ = 0.570, *t*(40) = 4.397, *p* < 0.001), faster mean reaction time (*b*_RTmean_ = −0.337, *t*(40) = −2.311, *p* = 0.026), higher attention (*b*_attention_ = 0.676, *t*(40) = 6.060, *p* < 0.001), and lower fidgeting (*b*_fidgeting_ = −0.678, *t*(40) = −6.036, *p* < 0.001) across models.

#### Learning Activity Measures.

We next examined whether similar EEG–behavior relationships were observed during naturalistic learning activities. As shown in Table 2, greater absolute alpha power during activities was associated with higher levels of attention, as indexed by active engagement (AE) and passive engagement (PE) during video watching (*b*_AE_ = 0.336, *t*(45)_AE_ = 2.407, *p*_AE_ = 0.020), online (*b*_PE_ = 0.341, *t*(45)_PE_ = 2.741, *p*_PE_ = 0.009), and in-person (*b*_PE_ = 0.445, *t*(45)_PE_ = 3.734, *p*_PE_ < 0.001) activities. In complement, greater absolute alpha power was associated with lower levels of fidgeting during online (*b* = −0.371, *t*(45) = −2.960, *p* = 0.005) and in-person (*b* = −0.443, *t*(45) = −3.348, *p* = 0.002) activities. In opposition to the laboratory task analysis, no consistent effects were observed for derived alpha ERMs during learning activities.

Consistent with the lab task model result, age showed significant associations with behavioral outcomes, with older age predicting higher passive engagement during online (*b* = 0.336, *t*(45) = 2.771, *p* = 0.008) and in-person (*b* = 0.312, *t*(45) = 2.667, *p* = 0.011), lower fidgeting during online (*b* = −0.288, *t*(45) = −2.363, *p* = 0.023), and lower off-task during online (*b* = −0.442, *t*(45) = −3.529, *p* < 0.001) and in-person (*b* = −0.326, *t*(45) = −2.324, *p* = 0.025) activities.

#### Neurocognitive Measures.

Finally, we examined whether EEG-extracted features in either lab or activity setting predicted academic performance and caregiver-reported neurocognitive functioning scores. For alpha power derived during the laboratory task, higher maintenance alpha power (*b* = 0.411, *t*(40) = 2.223, *p* = 0.032) and lower maintenance alpha ERM (*b* = −0.364, *t*(40) = −2.077, *p* = 0.044) predicted higher scores in WJ-IV PC (Table S7 in Supplementary Materials). In contrast, regression models predicting caregiver-reported survey measures were not significant for either lab task– or learning activity–derived alpha measures. Additionally, we note that age was a robust predictor of academic achievement, with older age associated with higher WJ-IV performance across models (Table S8 in Supplementary Materials; *b* > 0.75, *t*(45) > 8.42, all *p* < 0.001). As such, alpha power showed sparse associations with individual differences in academic achievement and neurocognitive functioning scores.

## DISCUSSION

The objective of this study was to test if alpha oscillations, a common metric of visual attention, correlate across laboratory versus naturalistic activities, as would be observed in a classroom. Thus, we tested if laboratory-derived measures capture the same mechanistic sources when deployed in more naturalistic environments. Results suggest that this is not necessarily the case, indicating the presence of what we call “measure heterogeneity” – whereby same measure may reflect different underlying processes depending on the context in which the measure is recorded. At the core of this inference is the failure to find significant correlations between alpha power recorded during a validated laboratory task, the spatial working memory (SWM) task, and absolute alpha power during learning activities. It is particularly notable that this lack of correlation was not due to the change in measure derivation, with event-related alpha modulations (ERMs) used to infer visual attention processes during the lab task and absolute measures during the learning activities. Moreover, absolute alpha power during the SWM task correlated significantly with the equivalent measure during learning activities, indicating preserved individual variability and internal consistency of the metric across different sessions of the experiment. Furthermore, the relation between baseline power and ERMs was replicated across activity and lab contexts, suggesting internal consistency in the absolute versus ERM power relationship. This pattern of results (summarized in Fig. 6) indicates that the metrics acquired during activity context may capture a process different or dissociable from their event-related derivatives during task stimulus processing.

There exist several candidates for these differences, though all appear related to attention outcomes. Namely, the regression analyses indicate that alpha power derivatives in both lab task and activities predict attention-related outcomes, and consistent with this both have been previously related to visual attention. Alpha power ERMs during the spatial working memory task have been repeatedly associated with visual encoding efficacy, including target enhancement or distractor suppression^17^, as well as associated occipital activity^21,22^ and connectivity^21,23^, and task performance^15,24^. The absolute measures observed during learning activities in the present study also show within-subject correlations with behavioral attention to the task^14^. The lack of correlations between the absolute and event-related processes thus most likely points to differing mechanisms that *both* contribute to behavioral indices of attention. In particular, whereas ERM measures likely index a transient process of visual encoding, the absolute measures may encompass influences of background or modulatory processes that also contribute to attention, such as arousal, motivation or motoric influences on neural activity – all of which have been previously shown to impact absolute alpha power^18,25,26^. Though in the present study we do not know which of these processes may have been at play, it is perhaps telling that the direction of alpha modulation is inconsistent in within- versus between-participant analyses. Namely, whereas alpha power systematically decreases during stimulus processing (a within-subject analysis) consistent with greater visual engagement, absolute alpha power is also lowest for individuals with greatest inattention and fidgeting behaviors. A parsimonious account is that in these individuals motor arousal is highest, contributing to greater baseline visual engagement and thus lowest alpha power. This could subsequently contribute to weaker ERMs in these individuals. If so, then the alternate factor that contributes to background measures of alpha power is motor arousal, which contributes to alpha power variability independently from visual encoding during a task^18^.

An implication of the results is that studies aiming to translate laboratory neural metrics to classroom learning context should consider measure heterogeneity. Though this observation is true for any measure in any experimental context, it is particularly important in the context of neuroscience of classroom learning because classroom environments – unlike laboratory settings – are subject to dynamic factors (e.g., noise, social interactions, fatigue) that impact modulatory systems like arousal. The results speak to longstanding discussions of the challenges connecting research in neuroscience and education (e.g., Varma et al.^27^), providing evidence that measures of attention in laboratory settings do not fully capture attention in the real world. In doing so, these results provide a tangible example of the methodological issues that are introduced when conducting neuroscientific research in complex, real-world environments. At the same time, the challenge highlights new opportunities, such as Identifying where laboratory measures diverge from real-world behavior to help guide the development of new paradigms, as well as measurement approaches that better capture learning as it unfolds in naturalistic settings. One proposed solution is the embedding of event-related structure within the classroom context, such as layering event-related visual stimuli in a teachers’ presentation screen^28^ or generating auditory noise events^29^, thus allowing for extraction of identical event-related responses in the classroom as in the laboratory. Another emerging approach is to derive sensory entrained responses from the neural signals, such as auditory stream related to a teacher’s voice^29^. Finally, a complementary approach is to ensure that neural measures are collected synchronously with physiological correlates, such as a heart rate, pupil diameter, or electrodermal activity, which may be critical in dissociating multiple contributing sources of behavior^30,31^.

The consideration that multiple sources, in addition to visual attention, contribute to inferred behavioral attention in a learning setting has theoretical implications for operationalization of attention in the classroom. It is notable that if the simple expectation – that visual-encoding alpha ERM correlates with individual differences in corresponding neural measures of attention or corresponding behavior during learning activities – had been supported, we would have a clear intervention target for improving attention. In that case, we could advise teachers to enhance classroom learning by ensuring that students visually encode content. The present results suggest a more complex reality. Visual attention in a student is one of several interacting processes that contribute to emergent behavioral attention and corresponding learning outcomes, and thus all must be considered. These data are well aligned with recognition of school engagement as a multidimensional phenomenon^32^.

The results also speak to the tension between the multidimensionality reflected in educators’ description of student attention^33^ and the nature of laboratory experiments of the same construct. This discrepancy may reflect differences between the goals of educational practice and neuroscientific research, and the tradeoffs inherent to conducting neuroscientific research in real-world settings^12^. Educator observations reflect the complexity of attention in the classroom – which involves a range of cognitive, physiological, motivational, and environmental factors that are difficult to isolate experimentally. Although it comes at a cost to ecological validity, the control afforded by tightly controlled laboratory paradigms is necessary for establishing a foundational understanding of neuroscientific measures before they can be meaningfully examined in the dynamic environments where learning occurs. To understand how educational settings impact brain and behavior, both perspectives are necessary^34^.

Collectively, the present study demonstrates that laboratory-task event-related neural measures, despite their high mechanistic specificity, do not necessarily correlate with between-subject differences in corresponding measures during naturalistic learning activities. We point to measure heterogeneity and multiple contributing mechanistic sources as a potential explanation, which implies that attention during classroom learning is unlikely to be driven by a single underlying mechanism. Instead, attentional engagement in educational contexts likely reflects the interaction of multiple processes – including multisensory processing, arousal, motivation, and motor activity – that together give rise to students’ observable behavior. From this perspective, improving attention in school is unlikely to depend on a single intervention targeting one mechanism. Rather, supporting student attention will likely require coordinated attention to instructional design, classroom environment, and individual differences in learners.

## METHODS

### Participants

The study enrolled 80 participants across two sites, approved by University of California, Los Angeles IRB (19–000139) and IRB of The University of Virginia (4956). All methods were performed in accordance with the relevant guidelines and regulations and in accordance with the Declaration of Helsinki, and in accordance with the approved protocols of the respective institutional review boards. We distributed flyers to recruit participants in the greater Los Angeles and Charlottesville area. The inclusion criterium was age 6 to 10 years old. The exclusion criteria were presence of serious medical or neurological illnesses likely to influence cognition or brain function (e.g., epilepsy, head trauma, or other neurologic disorders), and presence of clinically unstable conditions (e.g., suicidality, psychosis, mania, significant aggression, substance abuse). Informed consent was obtained from all participants’ legal guardians, and assent was obtained from all participants prior to data collection.

### Data Collection

#### Computerized Laboratory Tasks

After orientation and consent, participants performed three computerized cognitive tasks. These included eyes closed (EC), eyes open (EO), and spatial working memory (SWM)^19^, which we have previously used with children 6–14 years old. The EC and EO recordings were 1 min each. The screen was black during EC, and a snowflake was displayed in the center of the screen during EO. The EC and EO resting state were used as baseline reference conditions for assessing alpha power during the learning activities. After the learning activities, each participant also completed an executive function task (Hearts & Flowers; not included in current analyses)^35^. All computerized tasks were performed using Presentation^®^ software (Version 21; Neurobehavioral Systems, Inc., Berkeley, CA; www.neurobs.com).

#### Naturalistic Learning Activities

Participating children engaged in neuroscience-themed learning activities to learn about the brain and neuron. Drawing on the Individualized Student Instruction (ISI) framework^36^, we designed four learning activities to mimic real-world learning experiences. The activities varied in (a) instruction modality, and (b) instruction management. These manipulations are fully described in Chang et al.^14^ and were designed to test the effects of instructional context on engagement in learning. Briefly, *instruction modality* was varied among three teacher-led activities and differed in the amount of interaction: online video watching, online teacher instruction, in-person teacher instruction. The fourth activity, individual learning, was included to test the effect of *instruction management* (student-led). This condition was compared with the in-person instruction activity to evaluate if scaffolding due to teacher interaction increased engagement in learning. Each activity was designed to be 10-minute long and was delivered one-on-one by a trained instructor. Due to natural variations in the pace of interaction and the time needed to complete the individual learning task, the duration of activities varied among participants, with an average of 7.00 minutes for the online, 6.49 minutes for the in-person, and 8.42 minutes for the individual. The video watching activity was consistently 10-minutes because it was the same clip for all participants and did not involve any interaction. The four instruction contexts were presented in four different orders to avoid order effects.

#### EEG Recording

EEG was digitized at 250 Hz during all learning activities and the EO, EC and SWM tasks using the Smarting MOBI system (mBrain Train, Serbia) with a 24-channel saline cap (Greentek Pty Ltd, Wuhan, China). The EEG was recorded through Smarting Streamer (mBrain Train, Serbia). The impedance of each electrode was < 40 kW. Participants were encouraged to remain as still as possible during the EEG recording, but movement was not impeded or corrected.

### Data Processing

#### EEG Processing

We used custom scripts in MATLAB (The Mathworks, 2022) and EEGLAB^37^ to process EEG data and extract the oscillatory power. The native xdf EEG files were imported into EEGLAB, with electrode locations following a default head model template (standard-10–5-cap385.elp). The data were then filtered with a 0.1Hz high-pass filter and a 50Hz low-pass filter and then corrected to remove significant non-stationary artifacts using Artifact Subspace Reconstruction (ASR)^38^ with the burst criterion setting as 15. Then, recordings from computerized tasks and learning activities were merged, and TP9 and TP10 sensor data were removed as they contained excess muscle noise. Remaining outlier channels were detected using the flt_clean_channels.m function from the ASR toolbox, based on channel correlation profiles. The initial correlation threshold was set to 0.6, and the function iteratively identified bad channels based on their correlation with other channels. The threshold was dynamically adjusted to ensure that between 1 and 5 bad channels were detected. The bad channels were removed, and the EEG was referenced to average of all channels. The binary Infomax ICA^39^ was used to separate data into signal and noise sources. Then, the ICLabel machine learning algorithm^40^ was utilized to generate the maximal prediction confidence for each independent component (IC) that isolated artifact sources (i.e., muscle, eye, heart, line noise, and channel noise) from the signal. These artifacts were then subtracted from the EEG data prior to analysis.

Following cleaning, the continuous recordings of EC, EO, and learning activity data were segmented into 2-second, non-overlapping epochs. For SWM, the data were segmented into 11-second epochs; the stimuli occurred at time 0, with epochs spanning 2.5 seconds prior to stimulus onset and 8.5 seconds after stimulus onset. Excessively noisy epochs were identified based on the statistical properties of the time series, using a 5-standard-deviation threshold. Epochs were rejected in a single pass with the pop_autorej.m function in EEGLAB, with a rejection limit of up to 3% of total epochs (or at least one epoch if 3% was less than one). Additionally, to identify dataset outliers, we calculated the root mean square (RMS) power in the EC, EO, SWM, and activities epochs for each dataset. A dataset would be excluded from further analysis if: (i) the RMS was outside the interquartile range (IQR), (ii) > 50% of epochs were identified as noisy by the epoch rejection algorithm, or (iii) there were < 10 epochs remaining in the dataset. There were 51 participants included in the final analysis. Participants were excluded if: the participant was out of the age range during testing (N=1), the participant did not engage in the experiment (N=1), the parent/main caregiver did not respond to a diagnostic interview (N=3), data contained excessive line noise which could not be cleaned by the processing pipeline (N=2), EEG data loss > 50% (N=4), EEG data structure issues due to equipment failures (N=2), the trials < 10 in any of the baseline EEG recording, SWM or each activity (N=7) or the RMS (N=5) and SWM alpha ERM (N=4) were outside the IQR. The average number of retained epochs was 23 in EC (46 s), 22 in EO (44 s), 38 in SWM, 240 during video watching (480 s), 188 during the online activity (376 s), 166 during the in-person activity (332 s), and 210 during the individual activity (420 s). The average within-individual EEG data retention rate was 83.9%.

#### EEG Feature Extraction: Alpha Power

We used the power of alpha-band oscillations (8–12 Hz) to quantify visual attention engagement during the learning activities, based on its demonstrated negative correlation with engagement of occipital, parietal, frontal, and thalamic visuo-spatial attention networks^22,41,42^. As such, lowest alpha power would be expected in the most engaging conditions during the continuous learning activities, and during stimulus processing during the computerized tasks. Alpha power was calculated in two ways: as event-related modulations and as absolute power. This was done because the former is typically used in laboratory task settings to study event-related cognitive or perceptual processes whereas the latter is standard in recordings outside of laboratory task contexts, including clinical assessments.

##### Absolute Alpha Power.

To extract absolute alpha power, the power spectral density (PSD) for each frequency was calculated for each epoch using pwelch function of MATLAB without overlapping. Next, to extract power independently from spectral slope and offset, the extracted PSD was input into the spectral parameterization or specparam (formerly known as “Fitting Oscillations and One-Over-f” or FOOOF) toolbox^43^. This was done to extract alpha power (9–10 Hz) independently of spectral slope and offset. The parameters to fit the spectral distribution and identify peaks were: peak width limits = [1, 8], max number of peaks = 6, minimum peak height = 0.05, and aperiodic mode = “fixed”.

The 9–10 Hz alpha subrange was selected to represent alpha power in the current study because (1) a previous meta-analysis indicated that the peak alpha frequency was between approximately 9 and 10 Hz in child samples; and (2) the specparam algorithm detected that the central frequency of alpha power ranged from 9.09 to 10.05 Hz across two resting-state conditions, four activities, and SWM (during baseline, encoding, maintenance, and probe) in the selected channels. The averaged R^2^ was 0.98 and errors were 0.04 across the selected channels of the specparam fitting results.

##### Event-Related Modulations (ERMs).

Additionally, we extracted event-related modulations, derived from epoch time courses for each subject in the selected clusters. We focused our analyses on ERM in the alpha range (8–12 Hz) for consistency with prior literature. ERMs for SWM were computed following stimulus onset by dividing the frequency power by that obtained during the baseline (i.e., −1 to −0.5 s) and log-transforming the result to decibel units (dB). Frequencies between 8 and 12 Hz were averaged.

In contrast, because the learning activities did not contain discrete stimulus onsets, alpha ERM for the activities was computed relative to a resting-state condition. To compute alpha ERM for the learning activities, we first converted EO log(power) values to linear power using 10^EO log(power)^ to obtain EO power. Similarly, activity log(power) was transformed using 10^activity log(power)^ to obtain activity power for each condition (video watching, online, in-person, individual). Activity ERM was then computed relative to EO by converting the power ratio to decibel units: 10 ´ log _10_ (activity / EO).

*Clusters*. The extracted features were averaged across channels. Specifically, nine channels were selected for the alpha oscillation analysis, and they were grouped into three different clusters to increase signal-to-noise ratio (SNR) while covering the anterior to posterior scalp: F3, F4, and Fz were grouped as the frontal cluster; C3, C4, and Cz were grouped as the central cluster; O1, O2, and POz were grouped as the posterior cluster.

### Behavioral Outcomes

#### Video-Based Behavioral Coding

We used video-based behavioral coding to quantify attention-related behaviors throughout both activities and lab tasks. Behavioral coding served as a convergent outcome measure with respect to instruction context manipulations, and as validation for the interpretation of the EEG alpha metrics relative to observed behavior and vice versa. Datavyu (Datavyu Team, 2014), a free, open-source data coding tool, was used to perform all behavioral coding tasks. MATLAB was used to perform behavioral data analysis. Raw video-coded behavioral data was recorded in milliseconds and were converted to seconds and minutes in this report to facilitate interpretation.

Nine undergraduate students and the second author evaluated participants’ behaviors during activities and lab tasks using the Student Attention Tracking (SAT) protocol^44^, a behavioral coding protocol that integrates three existing behavioral coding systems for assessing student attention^45–47^. Averaged across lab tasks and learning activities, the inter-coder reliability was ICC = 0.947 (95% CI [0.936, 0.957]). SAT includes three behavioral categories divided into six codes: active engagement (AE), passive engagement (PE), macro fidgeting, micro fidgeting, passive off-task, and verbal off-task (Table S1 in Supplementary Materials). However, AE and PE were not applicable during SWM and Hearts and Flowers, as these tasks required continuous task-directed interaction with a laptop, limiting opportunities to distinguish between active and passive forms of engagement; therefore, participants were coded as engaged when they were performing the tasks.

For each behavioral code/category during each activity, a percentage was generated to capture the proportion of time exhibiting that behavior in relation to the total length of the activity. For example, during a 10-minute individual activity, if the participant had 7 minutes coded as actively engaged in making the neuron, then the percentage of AE during the individual activity would be (7 minutes /10 minutes) * 100% = 70.00%.

#### Task Performance

For the laboratory task, we also calculated several performance measures: accuracy, reaction time (RT) and reaction time variability expressed as coefficient of variation (CV).

#### Neurocognitive Assessments & Diagnosis

Finally, we also acquired outcomes related to neurocognitive functioning and ADHD symptomatology. ADHD symptom severity was assessed using caregiver-report measures, including the Conners 3–Parent Short Form^48^ and the Strengths and Weaknesses of Attention-Deficit/Hyperactivity Symptoms and Normal Behavior Scale (SWAN)^49^. Participants meeting clinical thresholds (T score >= 70) on the Conners 3 were referred for diagnostic evaluation by a licensed clinical psychologist using the Schedule for Affective Disorders and Schizophrenia for School-Age Children (KSADS)^50^. Caregivers also completed the Behavior Rating Inventory of Executive Function, Second Edition (BRIEF-2)^51^, and the Child Behavior Checklist (CBCL)^52^. Academic achievement and executive processing were assessed using five subtests from the Woodcock–Johnson IV (Form A)^53^.

### Statistical Analyses

#### Demographic and Participant Characteristics

The results of task performance and neurocognitive assessments are provided in the supplementary materials (Table S2).

#### Alpha Power Main Effects

To validate alpha modulation main effects, we analyzed alpha power across conditions within each condition separately. Namely, we tested alpha power changes across contextsduring learning activities, and across task phases and memory loads during the SWM task.

##### Learning Activities.

To examine differences in alpha power across activity contexts, a one-way repeated-measures analysis of variance (ANOVA) was conducted with activity (EC, EO, video, online, in-person, and individual) as the within-subject factor with age and symptom as covariates. When appropriate, post hoc pairwise comparisons were performed with correction for multiple comparisons. We expected alpha power to decrease with increased engagement of visual cortex, namely decreasing from EC, to EO, to video watching, to interactive activities.

##### Laboratory Tasks.

To assess task-related modulation of SWM ERM, a two-way repeated-measures ANOVA was conducted with load (load 1, load 3) and phase (encoding, maintenance, probe) as within-subject factors. Where applicable, follow-up comparisons were performed using least significant difference (LSD) tests to examine across load and phase. We expected alpha power to decrease during stimulus and probe processing, consistent with visual encoding processes. To further characterize phase-related consistency, we conducted one-sample t tests against zero for each phase and computed pairwise Pearson correlations among encoding, maintenance, and probe ERM values.

#### Alpha-Power: Activity versus Lab Task Correlations

To examine the consistency of alpha power variability during learning activities versus the SWM laboratory task, Spearman correlations were computed using the SciPy library^54^, with false discovery rate (FDR) correction applied using the statsmodels library^55^. Correlations were computed for both event-related derivatives and absolute alpha power, across activity and lab task data.

#### Alpha-Power: Relationships to Cognitive and Behavioral Outcomes

Finally, to support functional interpretation, we quantified the relationship of alpha power measures within each of activity and lab contexts to outcome measures. We conducted multiple regression analyses with EEG features (event-related & absolute alpha power, during both activity and lab task) as predictors and age (continuous), sex (categorical), and symptoms (categorical) included as covariates of no interest. A separate analysis was conducted for each outcome category: video-coded behaviors (engagement, fidgeting, off-task), derived factors based on caregiver-reported questionnaires (see below), and task performance.

Note that because the three alpha measures recorded during different phases of the lab task were strongly correlated for both alpha power and alpha ERM, maintenance and probe alpha power and alpha ERM were orthogonalized with respect to encoding alpha power and encoding alpha ERM, respectively, using linear regression. This procedure involved computing unstandardized residuals from regressions of maintenance and probe alpha measures onto the corresponding encoding alpha measures, thereby attributing shared variance to the temporally preceding encoding phase.

Finally, for the questionnaire-based outcomes, to reduce the dimensionality of the data, we first conducted a factor analysis using principal axis factoring. The analysis included symptom measures (Conners and SWAN attention scales), academic achievement (WJ), and caregiver-reported survey measures (CBCL and BRIEF-2), which assess symptoms and behavioral functioning. Model assumptions were tested using the Kaiser–Meyer–Olkin measure of sampling adequacy (> 0.6) and Bartlett’s test of sphericity (p < 0.001). Factors were rotated using Promax oblique rotation, selected based on the presence of factor correlations > 0.32^56^. The number of factors was selected based on eigenvalues greater than 1. This analysis identified four components: PC1 reflecting rule-breaking behavior, PC2 reflecting attention symptoms, PC3 reflecting anxiety scores, and PC4 reflecting somatic complaints. The details of the results are provided in the supplementary materials under Principal Component Analysis.

Across all analyses, statistical significance was set at α = 0.05. Descriptive analyses, factor analyses, and regression models were conducted using IBM SPSS Statistics (IBM Corp., 2022).

## Supplementary Material

This is a list of supplementary files associated with this preprint. Click to download.

- 260423R21manuscriptSciReportssupplementary.docx

## Figures and Tables

**Table 1.** Multiple Regression Models of Behavioral Measures on EEG Source Measures in Laboratory Setting

	*tb* (regression coefficient)										Model fit	
	Alpha_b_	Alpha_e_	Alpha_mê_	Alpha_pê_	ERM_e_	ERM_mê_	ERM_pê_	Age (yr)	Sex	Symptom	*R* ^2^_adj_	*F*
Accuracy	0.084	0.071	1.319	−0.272	1.318	**−2.100** *	0.759	**4.397** ***	−1.690	1.348	0.293	**3.068** **
RTmean	0.405	−1.464	0.349	1.165	1.221	0.358	−0.367	**−2.311** *	1.002	−1.067	0.106	1.591
RTCV	−0.481	−0.855	0.720	1.073	−0.056	−0.339	−1.824	−1.863	−0.082	−0.560	0.003	1.015
Attention	1.328	1.328	0.560	−1.398	1.562	−1.712	**2.719** **	**6.060** ***	0.062	0.211	0.477	**5.557** ***
Fidgeting	−0.855	−1.653	−0.012	0.900	−1.177	1.123	**−2.513** *	**−6.036** ***	−0.368	0.108	0.469	**5.413** ***
Off-task	−1.820	0.710	−1.871	1.914	−1.599	**2.277** *	−1.304	−1.531	0.955	−1.061	0.104	1.583

Note.

*** *p* < 0.001,

** *p* < 0.01,

* *p* < 0.05.

Alpha = background alpha power; ERM = event-related modulation; b = baseline; e = encoding; m⊥e = maintenance; p⊥e = probe; sex: 1 = male, 2 = female; symptom: 1 = high, 2 = low. RTmean = mean reaction time; RTCV = coefficient of variation of reaction time.

**Table 2.** Multiple Regression Models of Behavioral Measures on EEG Source Measures in Learning Activities

	*tb* (regression coefficient)					Model fit	
	Alpha	ERM	Age (yr)	Sex	Symptom	*R* ^2^_adj_	*F*
Video							
AE	**2.407** *	0.162	0.576	−1.056	−1.388	0.071	1.763
PE	1.055	0.661	1.687	1.610	1.290	0.084	1.913
Fidgeting	−1.424	−0.584	−1.553	**−2.113** *	−0.967	0.113	2.727
Off-task	0.179	−0.568	−1.020	1.147	−0.888	−0.034	0.671
Online							
AE	−0.784	−0.013	0.453	−1.290	0.049	−0.052	0.504
PE	**2.741** **	0.710	**2.771** **	−0.782	1.755	0.279	**4.873** **
Fidgeting	**−2.960** **	−0.459	**−2.363** **	1.192	−1.609	0.268	**4.666** **
Off-task	1.241	−1.292	**−3.529** ***	1.822	−1.508	0.231	**4.003** **
In-person							
AE	−0.048	−1.089	0.131	−0.014	−0.780	−0.069	0.351
PE	**3.734** ***	0.638	**2.667** **	−0.030	1.571	0.339	**6.131** ***
Fidgeting	**−3.348** **	−0.132	−1.669	0.228	−1.049	0.222	**3.852** **
Off-task	−0.921	−0.105	**−2.324** **	−0.538	−0.333	0.047	1.495
Individual							
AE	−0.278	0.313	0.207	0.243	1.795	−0.034	0.671
PE	0.842	−0.388	0.332	0.887	0.611	−0.057	0.462
Fidgeting	−0.945	−0.435	−0.214	−0.180	−1.944	0.004	1.043
Off-task	−0.036	0.438	−0.622	−1.583	−1.986	0.047	1.489

Note.

*** p < 0.001,

** p < 0.01,

* p < 0.05.

Alpha = background alpha power; ERM = event-related modulation; sex: 1 = male, 2 = female; symptom: 1 = high, 2 = low; AE = active engagement; PE = passive engagement.

## Data Availability

Data is available upon request.
